# The role of *Geobacter sulfurreducens* and *Geobacter* species in sustainable development

**DOI:** 10.1093/sumbio/qvag019

**Published:** 2026-05-15

**Authors:** Dario R. Shaw, Hari Ananda Rao, Pascal E Saikaly

**Affiliations:** Biological and Environmental Science & Engineering (BESE) Division, King Abdullah University of Science and Technology (KAUST), Thuwal 23955-6900, Saudi Arabia; Biological and Environmental Science & Engineering (BESE) Division, King Abdullah University of Science and Technology (KAUST), Thuwal 23955-6900, Saudi Arabia; Biological and Environmental Science & Engineering (BESE) Division, King Abdullah University of Science and Technology (KAUST), Thuwal 23955-6900, Saudi Arabia; Environmental Science & Engineering Program, King Abdullah University of Science and Technology (KAUST), Thuwal 23955-6900, Saudi Arabia

**Keywords:** *Geobacter sulfurreducens*, extracellular electron transfer, wastewater treatment, bioremediation, bioelectronics, biocatalysts

## Abstract

*Geobacter sulfurreducens* is a model electroactive bacterium whose remarkable extracellular electron transfer capabilities bridge microbiology, materials science, and sustainability. Over the past two decades, its metabolism has been harnessed for clean energy generation, wastewater treatment, and the development of environmentally friendly electronic materials. Its metabolic versatility aligns with multiple of the United Nations Sustainable Development Goals (SDGs). In environmental systems, *Geobacter* reduces toxic and radioactive metals to insoluble, stable forms, detoxifies organic pollutants, and mitigates methane emissions in anoxic soils, contributing to climate action and water quality (SDGs 6 and 13). At the materials frontier, *Geobacter* produces conductive protein nanowires that enable biological electronics and biosensors, opening new avenues for biodegradable, renewable electronic devices (SDGs 7 and 9). Moreover, its redox machinery allows ambient-temperature synthesis of catalysts, including single-atom and nanocluster metals, offering green alternatives to conventional high-energy synthesis processes (SDG 12). Taken together, *G. sulfurreducens* exemplifies how microbial metabolism can be engineered toward circular bioeconomy solutions that integrate pollution control, energy recovery, and material innovation. As research advances, this organism continues to define a new paradigm of “sustainable microbiology,” in which living systems serve as platforms for next-generation green technologies.

**Sustainability Statement**

*Geobacter sulfurreducens* exemplifies how microbial metabolism can advance clean water and sanitation (SDG 6). Its bioelectrochemical activity enables simultaneous wastewater treatment and energy recovery, transforming pollutants and toxic metals into stable, less harmful forms while ensuring water safety. Moreover, *Geobacter*-based biosensors provide sustainable tools for real-time water quality monitoring and contamination prevention. Through these capabilities, *G. sulfurreducens* links microbial processes to circular water management and resource recovery. Beyond water applications, *G. sulfurreducens* also contributes to SDGs 7, 9, 12, and 13, advancing renewable energy, green materials, and climate action.

* Geobacter sulfurreducens* is an anaerobic, model electroactive bacterium known for its ability to transfer electrons to insoluble substrates (e.g. metal oxides or electrodes) via extracellular electron transfer (EET) (Logan et al. 2019). Originally isolated from hydrocarbon-contaminated surface sediment, *G. sulfurreducens* belongs to a broader, physiologically diverse group of metal-reducing Geobacteraceae that inhabit anoxic sediments and subsurface aquifers, where they participate in carbon turnover, Fe(III) cycling, and contaminant transformation (Caccavo et al. 1994, Holmes et al. 2007). In these environments, *G. sulfurreducens* conserves energy by coupling the oxidation of electron donors, particularly acetate or hydrogen, to the reduction of extracellular electron acceptors such as Fe(III) minerals. It can also respire fumarate, malate, and elemental sulfur, highlighting the diversity of respiratory strategies available under oxygen-limited conditions (Caccavo et al. 1994). Changes in electron donor and acceptor availability further reshape its central metabolism, underscoring the plasticity of its respiratory pathways (Yang et al. 2010). The genome of *G. sulfurreducens* encodes an extensive repertoire of *c*-type cytochromes, two-component regulatory systems, and additional metabolic potential, including genes associated with aerobic, one-carbon, and complex carbon metabolism. These genetic features are consistent with its capacity to adapt to varying electron donors and acceptors, as well as fluctuating redox conditions (Methé et al. 2003). Although it was initially described as an obligate anaerobe, *G. sulfurreducens* can tolerate oxygen exposure and even grow at low oxygen concentrations, underscoring a greater physiological flexibility. This flexibility allows the organism to persist in diverse ecological environments, including fluctuating oxic-anoxic boundaries (Lin et al. 2004). Together, these physiological features place *G. sulfurreducens* within diverse ecological roles in anoxic environments, while also explaining why it has become such a useful model for sustainable biotechnology. Over the past two decades, this versatile metabolism has been harnessed for diverse sustainable applications addressing environmental pollution, clean energy generation, and green materials synthesis (Kumar et al. 2017, Logan et al. 2019, Guberman-Pfeffer et al. 2024). As society advances toward the United Nations Sustainable Development Goals (SDGs), *G. sulfurreducens* offers a distinctive biological platform linking microbial physiology with sustainable innovation (Fig. 1). In this perspective, we highlight the contributions of *G. sulfurreducens* to sustainability science and discuss emerging directions for the future, particularly focusing on several key domains: clean water and energy (SDGs 6 and 7), environmental bioremediation and greenhouse gas mitigation (SDGs 6 and 13), innovative sustainable bioelectronics (SDGs 9 and 12), and the development of green catalysts (SDG 12).

## Clean water, clean energy, and resource recovery

Conventional wastewater treatment consumes large amounts of electricity and generates significant greenhouse gas emissions, while billions still lack access to safe sanitation (Katuri et al. 2019). Achieving SDGs 6 (Clean Water and Sanitation) and 7 (Affordable and Clean Energy) demands technologies that treat water while recovering energy and resources from waste. In this context, *G. sulfurreducens* is recognized as an engine of sustainability, capable of converting pollutants into energy and waste into value (Katuri et al. 2019). Its metabolic capability allows it to channel electrons from organic oxidation to electrodes (Lovley and Holmes 2022), thereby integrating wastewater treatment, clean energy generation, and resource recovery. In microbial fuel and electrolysis cells (MFCs and MECs), *Geobacter*-dominated biofilms serve as self-assembling catalysts that oxidize acetate, propionate, and other volatile fatty acids in domestic wastewater while generating electricity (Hari et al. 2016). Laboratory and pilot studies report >80% organic removal and power densities of 1–2 W m⁻² (Logan et al. 2019). Because *Geobacter* transfers electrons directly rather than via mediators, these systems achieve high coulombic efficiency and long-term stability. As a result, wastewater treatment can shift from energy-intensive to energy-neutral or even energy-positive. When supplied with a small voltage, MECs generate H₂ or CH₄, achieving hydrogen yields up to 1.9 m³ H₂ m⁻³ day⁻¹ and energy recoveries approaching 80% (Call and Logan 2008). MECs treating real wastewater achieve up to 92% chemical oxygen demand (COD) removal when *Geobacter* dominates the anode (Bader et al. 2023). Integration with anaerobic digestion can further enhance CH₄ yield and operational stability (Hari et al. 2025). Hybrid systems, such as the anaerobic electrochemical membrane bioreactor (AnEMBR) and the two-stage MFC–AFMBR, achieve >95% COD and >99% total suspended solids removal under near-energy-neutral operation (Katuri et al. 2014, Ren et al. 2014). Collectively, these bioelectrochemical innovations enable the simultaneous production of clean energy, reusable water, and renewable fuels under ambient, self-regenerating, catalyst-free conditions, thereby contributing to the circular sustainability goals of SDGs 6 and 7. Importantly, scale-up efforts and pilot-scale implementations have demonstrated the technical feasibility of bioelectrochemical systems for wastewater treatment, achieving stable long-term operation and effective organic removal in microbial fuel cells (Liang et al. 2018, Babanova et al. 2020, Jadhav et al. 2022, Maye et al. 2025, Jafary et al. 2026) and microbial electrolysis cells (Cusick et al. 2011, Gil-Carrera et al. 2013, Heidrich et al. 2013, 2014, Cotterill et al. 2017, Chen et al. 2019, Huang et al. 2022, Guerrero-Sodric et al. 2023, 2025). For example, a 1000-L pilot MFC treating municipal wastewater operated continuously for over one year, achieving COD removal on the order of ~70%–90% while producing power densities of up to 125 W m⁻³ under optimized conditions (Liang et al. 2018).

* Geobacter sulfurreducens* typically operates within microbial communities rather than in isolation. Long-term, wastewater-fed bioelectrochemical reactors show that stable performance arises from cooperative consortia. In these communities, multiple *Geobacter* species coexist with exoelectrogens such as *Desulfuromonas acetexigens*, as well as with fermentative and methanogenic partners (Hari et al. 2016, 2017, Sapireddy et al. 2021, Tian et al. 2022). These consortia exhibit functional redundancy, with different species performing similar electroactive roles, conferring resilience to operational and environmental fluctuations (Sapireddy et al. 2021). Within these biofilms, *G. uraniireducens* complements *G. sulfurreducens* by rejuvenating decayed populations and suppressing prophage induction through interspecies signaling, thereby sustaining anode activity under stress (Ye et al. 2024). Such interactions create self-regulating electron-transfer networks that buffer the system against perturbations and extend reactor longevity. Furthermore, *Geobacter* species mediate direct interspecies electron transfer (DIET) with methanogens such as *Methanosaeta harundinacea* and *Methanosarcina barkeri*, accelerating methanogenesis and enhancing energy recovery (Rotaru et al. 2014, Yin and Wu 2019). For example, *G. metallireducens* can directly transfer electrons to *M. barkeri* when ethanol is used as the substrate (Rotaru et al. 2014), evidencing how DIET can substantially increase methane production rates and improve overall energy recovery. Conductive materials such as magnetite or biochar further strengthen these electrical partnerships (Yin and Wu 2019). Together, these interactions transform biofilms into adaptive, living electrical networks that underpin resilient wastewater treatment and carbon-neutral bioenergy generation, advancing SDGs 6 and 7.

Looking forward, *G. sulfurreducens* and related electroactive consortia hold great promise for advancing sustainable bioelectrochemical technologies. However, large-scale implementation remains constrained by mass transfer limitations, electrode fouling, and community instability under variable operational conditions. Addressing these challenges will require integrated biological and engineering innovations. For example, bioaugmentation of electroactive species, nutrient management strategies to sustain active populations, strain engineering for broader substrate utilization, and improvements in cytochrome expression and biofilm conductivity. These advancements can be further supported by optimized reactor operation, uniform biofilm distribution, and AI-driven process control. In this context, machine-learning and artificial intelligence approaches are emerging as powerful tools to manage the intrinsic complexity of *Geobacter*-based systems by enabling real-time, data-driven optimization. AI-driven controllers can dynamically adjust substrate dosing rates, electrode potentials, and hydraulic retention times in response to continuous sensor feedback. This helps maintain optimal electron transfer conditions and preventing performance losses associated with overgrowth or substrate limitation (Mahapatra et al. 2022, Li et al. 2023, Pardo et al. 2025, Mkilima 2026). Recent studies have demonstrated that machine-learning models can predict reactor performance and stability more accurately than traditional empirical approaches, allowing proactive operational adjustments that enhance power output, coulombic efficiency, and long-term stability (Malekmohammadi and Mirbagheri 2022, Li et al. 2023). More advanced frameworks incorporating reinforcement learning and digital twin models further enable self-optimizing bioelectrochemical reactors, in which operational strategies are continuously refined based on system behavior (Mkilima 2026, Wang and Ma 2026). Experimental demonstrations, such as AI-based optimization of nutrient and vitamin dosing in MFCs, have already shown improvements in electrical performance and treatment efficiency, highlighting the practical value of these approaches (Farahani et al. 2024). Together, the integration of AI-driven control with microbial and electrochemical engineering offers a promising pathway to overcome scale-up barriers and unlock the full potential of *Geobacter*-based bioelectrochemical systems for sustainable wastewater treatment and energy recovery.

### Environmental bioremediation and climate action

*Geobacter sulfurreducens* and other *Geobacter* species thrive in subsurface environments by coupling the oxidation of organic compounds to the reduction of insoluble metals (Shi et al. 2016, Kumar et al. 2017, Logan et al. 2019, Shaw et al. 2025). *Geobacter*’s ability to transfer electrons to metals is being harnessed to remediate polluted soils and waters in an environmentally friendly manner. A prime example is the bioremediation of toxic heavy metals and radionuclides (Holmes et al. 2002, Shelobolina et al. 2007, Cologgi et al. 2011, 2014, Holmes et al. 2015, Yun et al. 2016, Acevedo-González et al. 2025). *Geobacter sulfurreducens* can enzymatically reduce soluble, highly toxic metals such as uranium(VI) to insoluble forms (e.g. uranium(IV) oxide), effectively immobilizing these contaminants in mineral form (Lloyd 2003, Cologgi et al. 2014).This metabolic capability has been used to immobilize heavy metals and radionuclides in polluted aquifers, preventing uranium migration in groundwater (Anderson et al. 2003, Gregory and Lovley 2005, Williams et al. 2011, 2013, Dar et al. 2013). *In situ* bioremediation strategies that stimulate indigenous *Geobacter* populations, typically by adding acetate as an electron donor, have successfully precipitated uranium and contained other metal contaminants in the subsurface (Williams et al. 2011, 2013). Besides uranium, *G. sulfurreducens* can detoxify other metals, including chromium(VI), a carcinogenic groundwater pollutant, by enzymatically reducing it to the less mobile and less toxic chromium(III) form (Gong et al. 2018, He et al. 2019, Elmeihy et al. 2021, Dou et al. 2022, Lin et al. 2022). Similarly, *Geobacter* species can employ their reductive metabolism to precipitate other heavy metals (such as antimony and cobalt) and metalloids (such as arsenic), thereby helping to immobilize or remove these contaminants from the environment (Islam et al. 2005, Dang et al. 2017, Dulay et al. 2020, Xie et al. 2023). For example, arsenic is primarily sequestered through indirect reductive pathways mediated by biogenic iron minerals. Although *G. sulfurreducens* cannot enzymatically reduce arsenate (As(V)) for growth, its respiratory reduction of ferric iron (Fe(III)) generates Fe(II)-bearing precipitates, such as vivianite and magnetite, which can immobilize arsenic from solution (Islam et al. 2005). A similar indirect mechanism applies to antimony, as *G. sulfurreducens* is unable to enzymatically reduce antimonate (Sb(V)). In this case, the Fe(II) generated during Fe(III) reduction does not directly transform Sb(V). Instead, it promotes retention within newly formed iron mineral phases, such as magnetite and goethite (Xie et al. 2023). In contrast, cobalt detoxification proceeds predominantly through a more direct enzymatic pathway. Under cobalt stress, *G. sulfurreducens* activates cellular mechanisms that drive the reductive precipitation of Co(II) on the cell surface, resulting in the formation of cobalt-rich mineralized nanoparticles and a measurable removal of cobalt from the medium (Dulay et al. 2020). Through the combination of indirect Fe(II)-mediated processes and direct enzymatic reduction, *Geobacter* can effectively transform arsenic, antimony, and cobalt into immobilized forms, thereby reducing their mobility and toxicity in anaerobic environments. These capabilities directly advance SDG 6 by detoxifying water resources through environmentally friendly microbial processes. Metal reduction by *Geobacter* is not only a bioremediation tool but also a potential strategy for biomining, enabling the recovery of valuable metals from low-grade ores or waste streams. Studies have demonstrated that *G. sulfurreducens* can reduce and precipitate gold and silver ions into nanoparticles (Law et al. 2008, Tanzil et al. 2016, Chen et al. 2018, Liu et al. 2024), opening new avenues for recovering these precious metals from mining wastewater. This bio-precipitation approach provides a low-energy, nontoxic method for metal recovery from electronics waste or mine tailings. In contrast, traditional smelting or chemical reduction are energy-intensive and polluting. By enabling resource recovery from industrial waste, such microbial processes support circular economy principles (SDG 12) by reducing dependence on virgin mining and transforming waste into a resource. Beyond metals, *Geobacter* also contributes to the degradation of organic pollutants in anoxic environments. In hydrocarbon-contaminated soils and sediments, *Geobacter metallireducens* is a key organism that can anaerobically oxidize petroleum hydrocarbons (e.g. benzene) using Fe(III) oxides as electron acceptors (Anderson et al. 1998, Zhang et al. 2012, Castro et al. 2022). In contrast, *G. sulfurreducens* cannot oxidize aromatic hydrocarbons such as benzene, highlighting the metabolic diversity within the genus *Geobacter*. Field trials have shown that stimulating *Geobacter* activity *in situ*, for example, through nutrient or acetate addition, accelerates bioremediation in groundwater (Müller et al. 2017). Such stimulation often yields the concurrent reduction of metal contaminants (e.g. uranium) and organic pollutants, underscoring the broad environmental benefits of Fe(III)-coupled bioremediation processes (Müller et al. 2017). Together, these processes highlight *Geobacter*’s potential as a key microorganism for sustainable environmental cleanup strategies that align with global goals for clean water and pollution control.

Emerging evidence also highlights *Geobacter*’s potential role in mitigating greenhouse gas emissions, contributing to SDG 13 (Climate Action). In anoxic wetlands and paddy soils, members of the *Geobacteraceae* can outcompete methanogenic archaea when ferric iron is available (Lovley and Phillips 1987, Hori et al. 2010, Tang et al. 2016, Gabriel et al. 2020, Masuda et al. 2024). This can suppress methane production, which is a significant climate benefit because methane is a potent greenhouse gas. Studies show that adding Fe(III) (e.g. ferrihydrite) to anaerobic soils stimulates *Geobacter* activity while inhibiting methanogens (Lueders and Friedrich 2002, Masuda et al. 2024, Yu et al. 2024). This can result in a bloom of *Geobacteraceae* and a corresponding decline in methane generation (Lueders and Friedrich 2002, Masuda et al. 2024, Yu et al. 2024). Both *Geobacter* and methanogens utilize fermentation-derived substrates such as acetate, but when Fe(III) is present, *Geobacter* preferentially uses these substrates to reduce iron, diverting the carbon flow from CH₄ to CO₂. Managing soil redox conditions or applying iron oxide amendments to promote *Geobacter* activity could therefore serve as a nature-based climate mitigation strategy. While this remains speculative, such findings highlight the potential of *Geobacter* as a microbial tool for greenhouse gas mitigation (Li and Zhou 2020). In practice, the amount of Fe(III) required to divert acetate consumption away from methanogens is high. Stoichiometrically, ~8 moles of Fe(III) are required per mole of methane suppressed, and delivering iron amendments at scale presents practical challenges. Thus, this approach remains highly speculative at present. Nonetheless, these findings illustrate the principle that stimulating Fe(III) reducers such as *Geobacter*, whether through natural soil treatments or engineered approaches, can suppress methane generation and highlights their potential role in greenhouse gas mitigation.

Future efforts should focus on scaling up *Geobacter*-based technologies and transferring lab-scale findings to real-world applications. Progress from bioremediation will require coordinated efforts across disciplines, including engineering, environmental microbiology, and materials science. Key challenges to address include maintaining microbial activity under variable environmental conditions, optimizing electron transfer across interfaces, and integrating biological systems into existing infrastructure. With continued interdisciplinary innovation, *Geobacter* could become a cornerstone organism in sustainable environmental biotechnology.

### Innovative bioelectronic technologies

One of the most remarkable metabolic features of *G. sulfurreducens* is its ability to transfer electrons extracellularly via microbial nanowires (Gu et al. 2021, 2023, Liu et al. 2021, Wang et al. 2022, Guberman-Pfeffer et al. 2024, Portela et al. 2024, Schwarz et al. 2024). These microbial nanowires allow biological systems to interface with electronic devices and fuel breakthroughs in sustainable electronics and biosensors (SDGs 6, 7, and 9) (Guberman-Pfeffer et al. 2024, Li et al. 2025). *Geobacter sulfurreducens* produces nanowires containing conductive *c*-type cytochromes (e.g. OmcS, OmcE, and OmcZ), which are inherently conductive and can transport electrons over micrometer distances (Gu et al. 2021, 2023, Wang et al. 2022, Portela et al. 2024). Cryo-EM structural studies of *Geobacter* nanowires revealed a unique protein fold tightly wrapping around a heme chain, enabling metal-like conductivity in a fully biological polymer (Gu et al. 2021, 2023, Wang et al. 2022). These nanowires are composed of polymerized *c*-type cytochromes. Each nanowire contains covalently bound heme groups that stack within the filament, forming a continuous electrical conduit along the fiber (Gu et al.^2021^, ^2023^, Wang et al. 2022). This “heme wire” architecture explains how electrons can flow over micrometer distances. The polymerization process of nanowires in *G. sulfurreducens* resembles the formation of organic conductive polymers (Malvankar and Lovley 2012, Li et al. 2025). Because these filaments are composed entirely of biological elements and are fully biodegradable, they represent an environmentally friendly alternative to synthetic nanostructures in electronics. Electrically conductive pili (e-pili), assembled from type IV pilin monomers, have been proposed as essential conduits for long-range electron transfer in *Geobacter* biofilms (Lovley and Walker 2019, Lovley and Holmes 2020, Liu et al. 2021). In parallel, structural and functional analyses have highlighted cytochrome-based nanowires as key contributors to extracellular electron transport in *G. sulfurreducens* (Gu et al. 2021, 2023, Wang et al. 2022, Portela et al. 2024). Accordingly, the mechanism underlying long-range electron transfer remains under active investigation, and the relative contributions of pilin-based structures, cytochrome nanowires, and redox-active networks within the biofilm matrix remain a matter of ongoing debate. Research continues to evaluate the role and nature of nanowires under different physiological, environmental, and engineered conditions, with increasing attention to context-dependent mechanisms and the potential regulation of electron transfer pathways in response to changing environmental and engineered parameters.

Beyond their biological role, *Geobacter* nanowires have been harvested and repurposed as sustainable electronic materials (Guberman-Pfeffer et al. 2024, Li et al. 2025). Unlike traditional nanowires or carbon nanotubes that require toxic chemicals for functionalization, protein nanowires are entirely biological, biodegradable, and can be genetically engineered (Lekbach et al. 2023). This approach eliminates the need for synthetic reagents and aligns with green electronics principles (SDG 9: environmentally sound technological development). For instance, incorporating e-pili nanowires into polymers yields conductive, biologically compatible composites that can serve as transient electronics or implantable bioelectrodes (Sun et al. 2018, Sonawane et al. 2025). Thin films of *Geobacter* protein nanowires have also been developed as wearable humidity sensors that rapidly and reversibly detect moisture changes (Liu et al. 2020a, 2020b, 2022). These films can also continuously generate electricity from ambient humidity, producing approximately 0.5 V and 17 μA cm⁻² from a 7 µm film (Liu et al. 2020b). A 2 µm thick film of purified *Geobacter* nanowires integrated onto a flexible substrate responded to human breath and skin humidity with high sensitivity, demonstrating applications in health monitoring (Liu et al. 2020b). Because these protein wires are produced from renewable feedstocks (bacterial cultures) and operate at ambient conditions, such devices represent green wearable electronics. They are thin, flexible, nontoxic, and biodegradable, require minimal energy to fabricate, and can operate under a wide range of environmental conditions (Guberman-Pfeffer et al. 2024, Li et al. 2025). In parallel, recent electronic and sensing devices have also been fabricated using electrically conductive nanowires (i.e. e-pili) that are heterologously expressed and mass produced in *E. coli*, demonstrating an alternative and scalable route for nanowire-based device fabrication, particularly for engineered sensing platforms (Lekbach et al. 2023, Sonawane et al. 2025).

*Geobacter* biofilms have also been reported to exhibit photoconductivity (Neu et al. 2022). A recent study revealed that OmcS nanowires in living biofilms can act as intrinsic photoconductors (Neu et al. 2022). When exposed to light, these nanowires exhibited a 100-fold increase in current and ultrafast (∼200 fs) heme-to-heme electron transfer, allowing *Geobacter* to harness light energy despite lacking chlorophyll. Essentially, these biofilms behave as living photoconductors, a property that could be exploited for solar-powered biocatalysis or light-responsive biosensors. Unlike traditional semiconductors, these biological wires do not require toxic dopants and do not suffer from photocorrosion (Neu et al. 2022). This finding opens the possibility of integrating microbial systems with optoelectronic devices. Future research could focus on engineered biofilms that route electrical signals in response to optical inputs, forming the foundation for biologically based information processing.

*Geobacter sulfurreducens* has also enabled the development of novel biosensors for water quality monitoring, further supporting SDG 6. *Geobacter*-based biosensors exploit the organism’s ability to generate electrical current from organic matter as a real-time indicator of water chemistry (Li et al. 2025). In these devices, *Geobacter* biofilms at the anode oxidize pollutants or bioavailable organics and release electrons, producing a measurable current (Commault et al. 2016, Estevez-Canales et al. 2018, Li et al. 2021, 2025, Olias and Di Lorenzo 2021, Huerta-Miranda et al. 2023). Changes in current output serve as an electronic signal of water quality. *Geobacter*-based sensors have been used to detect a wide range of parameters, from biochemical oxygen demand (BOD) to heavy metals, by correlating metabolic activity with electrical output (Commault et al. 2016, Estevez-Canales et al. 2018, Li et al. 2021, Olias and Di Lorenzo 2021, Huerta-Miranda et al. 2023, Li et al. 2025). Because *Geobacter* preferentially metabolizes simple organics such as acetate and thrives in electrode-associated biofilms, it provides a stable bioreceptor for these sensors. These living biosensors are highly sensitive and reagent-free, making them attractive for sustainable, low-cost environmental monitoring. Recent prototypes include self-powered sensors suitable for deployment in remote or resource-limited settings (Olias and Di Lorenzo 2021). Such technologies align with SDG 6 targets for affordable and environmentally friendly water monitoring. In the future, networks of *Geobacter*-powered biosensors could continuously monitor water quality and wastewater systems, providing early warnings and guiding remediation within sustainable sanitation infrastructures.

In essence, *Geobacter* nanowires act as biological nanoscale cables and sensors. These properties are inspiring the design of living electronics, hybrid systems in which biological components self-assemble into electrical circuits. Future efforts should focus on integrating *Geobacter* nanowires into wearable sensors and biodegradable electronic systems. Although significant engineering challenges remain, the convergence of microbiology and electronics in *G. sulfurreducens* foreshadows a new era of sustainable technology, one where circuits can be grown rather than manufactured. Looking ahead, bridging *Geobacter* research with synthetic biology could further enhance its utility. Genetic modifications may improve its tolerance to harsh waste streams or optimize nanowire production for bioelectronic applications. Scalability remains a challenge, and translating laboratory demonstrations such as nanowire-based devices to industrial scale will require innovation in bioprocess design and production.

### Development of sustainable catalysts

The electroactive capabilities of *G. sulfurreducens* can be harnessed to synthesize green catalysts for energy and environmental applications (Kalathil et al. 2019, 2020, Pedireddy et al. 2021, Jimenez-Sandoval et al. 2023, 2024, Bajracharya et al. 2024). Conventional synthesis methods for catalysts used in reactions such as water splitting, CO₂ reduction, or pollutant degradation are often energy-intensive, rely on toxic reagents and costly precious metals, and generate significant waste (Bajracharya et al. 2024). In contrast, *Geobacter* offers a biological route under ambient conditions, eliminating hazardous chemicals and minimizing energy input. *Geobacter sulfurreducens* functions as a bio-based platform for catalyst synthesis, leveraging its EET machinery to nucleate and grow catalytically active materials under mild conditions (Kalathil et al. 2019, 2020, Pedireddy et al. 2021, Jimenez-Sandoval et al. 2023, 2024, Bajracharya et al. 2024). Through this mechanism, the bacterium can reduce dissolved metal ions and deposit them as catalytically active species directly on its cell surface. This microbial-mediated synthesis occurs at ambient temperature and pressure, in water, and typically without the need for additional reducing agents or stabilizers (Kalathil et al. 2019, 2020, Pedireddy et al. 2021, Jimenez-Sandoval et al. 2023, 2024, Bajracharya et al. 2024). Unlike conventional nanomaterial synthesis, which often requires toxic ligands or extreme conditions, the *Geobacter* approach eliminates hazardous reagents and aligns with the principles of green chemistry. This strategy contributes to SDG 12 by reducing waste and energy use in catalyst manufacturing and supports a circular economy in which biomaterials and metal waste streams can be repurposed into valuable catalytic products.

A series of studies have demonstrated the versatility of *G. sulfurreducens* as a living catalyst factory. Kalathil et al. (2019) showed that *Geobacter* can reduce graphene oxide (GO) to reduced graphene oxide (rGO) while simultaneously doping it with heteroatoms, creating a hybrid electrocatalyst for oxygen evolution reaction (OER) (Kalathil et al. 2019). In this process, *G. sulfurreducens* served a dual role: its metabolism provided electrons to chemically reduce and modify graphene, while active elements such as Fe, Cu, N, P, and S became embedded in the graphene matrix (Kalathil et al. 2019). The resulting *Geobacter*-rGO hybrid exhibited OER performance comparable to commercial IrO₂, while relying only on abundant, nonprecious elements (Kalathil et al. 2019). This work exemplifies how *Geobacter* can assemble functional catalytic materials from simple precursors. Building on this foundation, Pedireddy et al. (2021) reported a method to synthesize single-atom metal catalysts (SACs) by incubating *G. sulfurreducens* with metal precursors under anaerobic conditions (Pedireddy et al. 2021). This ambient-temperature process produced single atoms of Fe, Ir, Pt, Ru, Cu, and Pd anchored on the cell surface without the need for thermal treatment (Pedireddy et al. 2021). The bacterium’s electron transfer machinery reduces metal ions and anchors individual metal atoms to its cell envelope, likely coordinated by native biomolecules such as cytochrome hemes. Remarkably, the resulting Ir single-atom catalyst exhibited exceptional electrocatalytic performance for water splitting, outperforming commercial IrO₂ for the OER and matching commercial Pt/C for the hydrogen evolution reaction (HER) (Pedireddy et al. 2021). This study established *Geobacter* as a scaffold for single-atom catalysis, where simply changing the dissolved metal source produces different SACs on the bacterial surface. Traditionally, fabricating single-atom catalysts requires high-temperature pyrolysis or vacuum deposition; with *Geobacter*, the same outcome can be achieved using a microbial culture at room temperature (Pedireddy et al. 2021). Expanding this approach to nonprecious metals, Jimenez-Sandoval et al. (2024) demonstrated the biological synthesis of nickel single-atom catalysts using *G. sulfurreducens*, an important advance given nickel’s role in alkaline water electrolysis (Jimenez-Sandoval et al. 2024). Synthesizing Ni single-atom catalysts by conventional means is challenging because Ni tends to aggregate, but *Geobacter* naturally chelates Ni on its surface (Jimenez-Sandoval et al. 2024). The resulting Ni–*Geobacter* catalyst exhibited activity for the HER and OER comparable to that of noble metal catalysts (i.e. Pt for HER and IrO₂ for OER) (Jimenez-Sandoval et al. 2024). X-ray spectroscopy revealed that Ni atoms were coordinated by nitrogen in *Geobacter*’s cytochromes, creating atomically dispersed catalytic sites stabilized by the natural heme environment (Jimenez-Sandoval et al. 2024). The Ni-loaded cells exhibited long-term stability under electrolysis conditions, further emphasizing the advantages of this biosynthetic approach. From a green chemistry perspective, this microbial process avoids the toxic reducing agents and surfactants used in traditional Ni nanoparticle synthesis, generates no hazardous waste, and operates entirely in aqueous media. It exemplifies sustainable innovation by merging microbiology with catalysis, using living cells to fabricate advanced materials.

The use of *G. sulfurreducens* in catalyst synthesis is still expanding. Future research should explore *Geobacter*-mediated synthesis of catalysts for CO₂ reduction, ammonia synthesis, and other key reactions. Genetic engineering of *Geobacter* to express modified cytochromes that bind metals more effectively or nucleate specific nanostructures such as bimetallic alloys or patterned arrays could yield designer biocatalysts with tunable compositions and architectures. Another promising research direction is to evaluate *Geobacter* biofilms as dynamic, regenerating catalysts. Because the cells remain metabolically active, electrodes coated with living *Geobacter* could self-regenerate catalytic sites through continuous biomineralization during operation, dramatically extending catalyst lifetime. By merging microbiology, materials science, and electrochemistry, *Geobacter sulfurreducens* is establishing a new paradigm for sustainable catalyst production that emphasizes low environmental impact, resource efficiency, and high performance. This biomimetic approach aligns with the principles of green chemistry: it uses water-based microbiological cultures, avoids toxic reducers and surfactants, and operates at ambient pressure and temperature. Using non-noble metals such as Ni and Fe further supports circular economy goals since these metals are abundant and easily recyclable. Ultimately, *Geobacter*-based catalyst production fits naturally within the circular bioeconomy, enabling the recycling of metals and biomass for sustainable industrial use.

## Conclusions

*Geobacter sulfurreducens* stands as a model organism for sustainable biotechnology, demonstrating how microbial metabolism can directly contribute to clean energy, environmental remediation, and green materials production. Its unique EET mechanisms have been harnessed for heavy metal bioremediation, greenhouse gas mitigation, and the development of conductive nanowires that enable bioelectronic and sensing applications. Recent advances have revealed the molecular basis of its conductive cytochromes and nanowires, particularly OmcS, OmcE, and OmcZ, opening the door to the design of bioengineered nanowires with enhanced performance. Moreover, *Geobacter*-mediated synthesis of catalysts, including single-atom and nanocluster metals, establishes a low-energy, non-toxic route for producing materials essential2 to renewable energy systems. Looking forward, efforts should focus on translating these laboratory discoveries into field-scale technologies for bioremediation, bioelectronics, and catalyst production. Key priorities include optimizing microbial performance under fluctuating environmental conditions, improving electron transfer efficiency across biomaterial interfaces, and integrating *Geobacter*-based processes into existing industrial and environmental infrastructures. Advances in genetic engineering, synthetic biology, and bioprocess design will be pivotal to enhance nanowire conductivity, catalyst yield, and system scalability. Ultimately, the convergence of microbiology, materials science, and engineering will consolidate *G. sulfurreducens* as a key organism in the circular bioeconomy, providing sustainable solutions that advance global goals for clean water, renewable energy, responsible production, and climate action.

**Figure 1:**
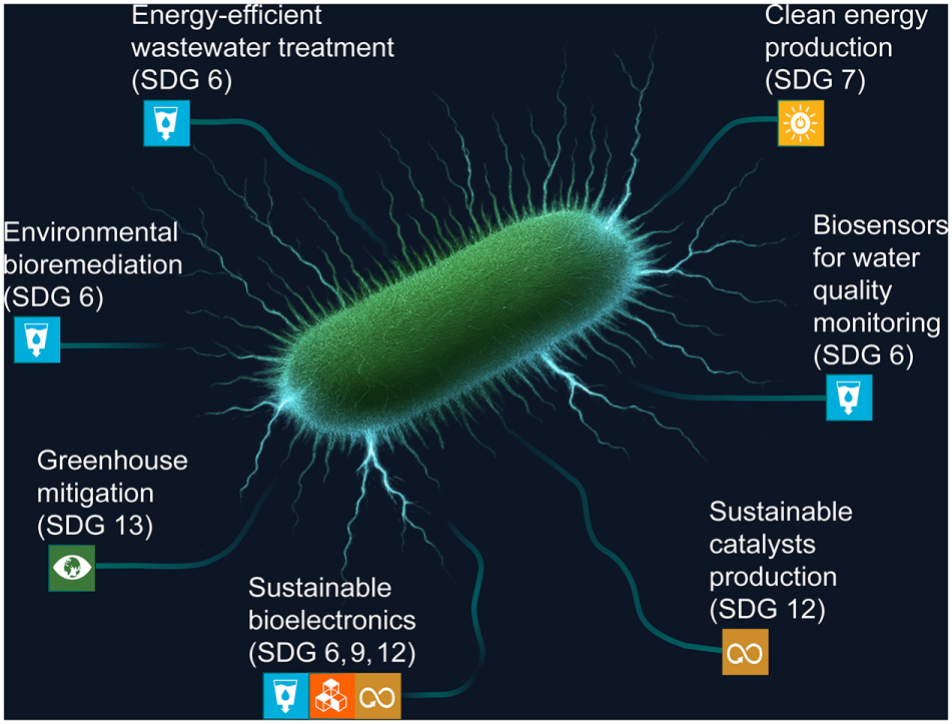
Sustainable applications of *G. sulfurreducens* linked to the United Nations SDGs. *Geobacter sulfurreducens*’ EET capabilities link microbiology, materials science, and sustainability. Its metabolism underpins diverse applications contributing to multiple SDGs: energy-efficient wastewater treatment and environmental bioremediation (SDG 6: Clean Water and Sanitation); greenhouse gas mitigation (SDG 13: Climate Action); clean energy generation (SDG 7: Affordable and Clean Energy); biosensing for water quality monitoring (SDG 6: Clean Water and Sanitation); sustainable bioelectronics (SDGs 6, 9, and 12: Clean Water and Sanitation; Industry, Innovation and Infrastructure; responsible Consumption and Production); and green catalyst production (SDG 12: Responsible Consumption and Production).

## Data Availability

All relevant data are contained within this article.
